# Multivariate statistical models of metabolomic data reveals different metabolite distribution patterns in isonitrosoacetophenone-elicited *Nicotiana tabacum* and *Sorghum bicolor* cells

**DOI:** 10.1186/2193-1801-3-254

**Published:** 2014-05-20

**Authors:** Ntakadzeni E Madala, Lizelle A Piater, Paul A Steenkamp, Ian A Dubery

**Affiliations:** Department of Biochemistry, University of Johannesburg, Auckland Park, 2006 South Africa; BioSciences division, CSIR, Pretoria, 0001 South Africa

**Keywords:** *Nicotiana tabacum*, *Sorghum bicolor*, Metabolomics, Isonitrosoacetophenone, PCA, HCA, OPLS-DA, SUS, Metabolic trees, UHPLC-Q-TOF-MS

## Abstract

Isonitrosoacetophenone (INAP, 2-keto-2-phenyl-acetaldoxime) is a novel inducer of plant defense. Oxime functional groups are rare in natural products, but can serve as substrates depending on existing secondary pathways. Changes in the metabolomes of sorghum and tobacco cells treated with INAP were investigated and chemometric tools and multivariate statistical analysis were used to investigate the changes in metabolite distribution patterns resulting from INAP elicitation. Liquid chromatography combined with mass spectrometry (UHPLC-MS) supplied unique chemical fingerprints that were generated in response to specific metabolomic events. Principal component analysis (PCA) together with hierarchical cluster analysis (HCA) and Metabolic Trees were used for data visualization. Orthogonal projections to latent structures discriminant analysis (OPLS-DA) and shared and unique structure (SUS) plots were exploited in parallel to reveal the changes in the metabolomes. PCA indicated that the cells responded differentially to INAP through changes in the metabolite profiles. Furthermore, HCA and Metabolic Trees showed that INAP induced metabolic perturbations in both cell lines and that homeostasis was re-established over time. OPLS-DA-based shared and unique structure (SUS) plots confirmed the results and revealed differences in the metabolites distribution patterns between tobacco and sorghum cells. Chemometric analyses of metabolomic data offers insight into changes in metabolism in response to chemical elicitation. Although similar, the response in sorghum cells was found to be more consistent and well-coordinated when compared to tobacco cells, indicative of the differences in secondary metabolism between cyanogenic and non-cyanogenic plants for oxime metabolism.

**Electronic supplementary material:**

The online version of this article (doi: 10.1186/2193-1801-3-254) contains supplementary material, which is available to authorized users.

## Background

Metabolomics is an unbiased approach aimed at measuring the metabolite content of a cell, tissue or organism under a given physiological status (Nicholson et al. 1998;Oliver et al. 1998). It is the analyses of these metabolites which lead to a comprehensive understanding of the unique chemical fingerprints that result from specific cellular processes (Theodoridis et al. 2011) and, as opposed to the analysis of genes or proteins, allows a thorough elucidation of the phenotypical characteristics of living systems. Metabolomics has recently found significant applications in many fields such as responses to environmental stresses (Lin et al. 2006;Viant 2007), studying global effects of genetic manipulation, nutrition and health (Van der Greef et al. 2004;Goodacre 2007) and, most importantly, in plant studies (Kopka et al. 2004;Weckwerth and Morgenthal 2005;Hall 2006;Kim et al. 2010;Tugizimana et al. 2013,2014).

Biochemical processes are intrinsically dynamic and for metabolomic studies the choice of sample preparation, analytical platform and subsequent data analyses are of critical importance (Dunn et al. 2005;Lu et al. 2008;Kim et al. 2010;Olivier and Loots 2012;Allwood and Goodacre, 2010). In the current study, ultra high performance liquid chromatography coupled to mass spectrometry (UHPLC-MS) was used for metabolite data acquisition based on its technological advances and ability to analyze a broad spectrum of metabolites of different polarities (Plumb and Wilson 2004;Allwood and Gooadacre, 2010). UHPLC-based methods detect more metabolites and generates more data output (Wilson et al. 2005). Data analysis is an essential step during metabolomic studies, since meaningful information needs to be extracted from structurally complex datasets (Robertson 2005). Here, both univariate and multivariate analyses can play complementary roles (Saccenti et al. 2014). It is therefore important that the design of metabolomic experiments is well considered so that valid and reproducible results can be converted into biological knowledge.

In contrast to transgenic approaches where genes encoding defense components of one plant can be transferred to another to result in new metabolite capabilities (Bak et al. 2000), novel metabolites can also be generated by supplying xenobiotic precursor molecules that are capable of being recognized by biocatalysts or a biological system already present in the plant (Madala et al. 2012a) through a process of biotransformation (Omiecinski et al. 2011). Novel enzyme-substrate combinations *in vivo* can lead to the biosynthesis of new, natural product-derived compounds (Pollier et al. 2011). We have previously reported that isonitrosoacetophenone (INAP), a precursor/activity determining motif of citaldoxime, a phytoalexin and anti-oxidant stress metabolite (Dubery et al. 1988,1999), is metabolized and bio-converted in tobacco cells (Madala et al. 2012a).

Here, chemometric data analyses, including multivariate data analysis (MVDA) models such as Principal Component Analysis (PCA), Hierarchical Cluster Analysis (HCA), and the Shared and Unique Structures (SUS) plot generated by Orthogonal Projections to Latent Structures Discriminant Analysis (OPLS-DA), were used to investigate the global effect of INAP on two metabolically distinct cell lines from *Nicotiana tabacum* (Solanaceae) and *Sorghum bicolor* (Poaceae). The HCA- and SUS plots as well as Metabolic Trees, were used together to decipher the metabolite distribution pattern responses at different time intervals, which allowed differentiations to be drawn with regard to the metabolism of oximes in the two cell lines that are non-cyanogenic and cyanogenic respectively. The results are discussed against the background of the emerging concept of dynamic metabolons (Møller 2010;Neilson et al. 2013).

## Results and discussion

As the aim was to focus on changes of intracellular metabolites and their coordinated or complementary behavior in relation to INAP metabolism, a MVDA approach was followed to analyse the UHPLC-MS -generated data (Saccenti et al. 2014). Metabolomic studies result in highly complex data which are spread in multi-dimensional space and dimensionality reduction is an important first step for pre-processing such data so as to extract meaningful information (Yamamoto et al. 2009). MVDA techniques such as the descriptive PCA and HCA (dimensionality reduction and pattern recognition methods), and explicative/predictive models like OPLS-DA, are used to achieve this (Fiehn et al. 2000;Jolliffe 2002;Wiklund et al. 2008;Saccenti et al. 2014).

### Principal component analysis

PCA, an unsupervised model, is an orthogonal linear transformation of possibly correlated variables into a smaller number of uncorrelated variables called principal components (PCs), where the greatest variance within the data by any projection is explained on the first coordinate (PC1) and the least variance is explained/projected by subsequent PCs (Jolliffe 2002). PCA and other reduction models thus convert the data into score plots, visual representations where data from different biological backgrounds are separated into distinct clusters. Samples that group together represent a specific “metabolic phenotype” (Fiehn et al. 2000).

**Figure 1:**
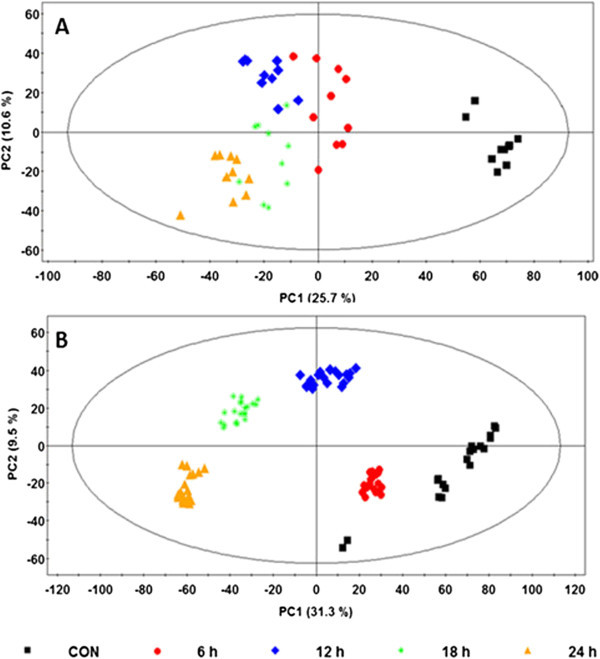
**PCA score plots showing the different clusters of samples from tobacco and sorghum at different time intervals following elicitation**. Mid-polar metabolites were extracted from INAP-treated tobacco **(A)** and sorghum **(B)** cell suspensions at different time intervals as represented by different colours and symbols on the plot (key for different time intervals is indicated). Model validation gave R^2^ *X* = 0.6 and *Q* ^2^_(cum)_ = 0.50 for the tobacco model (4 PCs) and R^2^ *X* = 0.64 and *Q* ^2^_(cum)_ = 0.48 for the sorghum model (7 PCs).

From the PCA score plots (Figure 1), it can be seen that INAP induced metabolic perturbations in both cell lines. The samples originating from the treated and non-treated cells clustered in different areas in the plots. As expected, the plot shows that variation between the different biological/treatment groups is more pronounced on the PC1 which counts for the highest variation in the models. The corresponding PC1 (describing the variation between groups) from the two plots was found to be 25.7% and 31.3% respectively, and PC2 (which describes the variation within the groups) was 10.6% and 9.5% for the tobacco and sorghum models respectively. However, the difference amongst all the treatment time intervals was found to be not as distinct, especially when the later time points (12, 18, and 24 h) are considered. From these plots (Figure 1A and B) it is clear that although the 6 h time point exists as a distinctive cluster, it possesses less variation from the control as compared to the other time points and could be due to the fact that the metabolic response(s) are still minimal at such an early time interval. In cases such as the one where variation amongst the later time points is less prominent (due to the fact that the separation of data clusters is not as clearly defined), measures need to be taken to overcome this. Since the presentation of the data in PC scores space is the result of an unsupervised method, this only shows a qualitative separation and the degree of separation between data clusters is not quantitatively addressed by the score plots (Werth et al. 2010). Thus, the basic statistical question regarding significant differences between the clusters is not addressed by PCA score plots even though the visualization represents a qualitative clustering due to metabolic differences. As such, subsequent plots, including the loading scatter plot, are used to evaluate the causative factors which result in different clustering on score plots (Yamamoto et al. 2009). From here, metabolites that are either up- or down-regulated can be selected to further evaluate their degree of significance across the clusters which they influence. The loading scatter plots (not shown) corresponding to these PCA score plots revealed metabolites or signatory biomarkers (*m/z* ions), which can be assumed to be influential of the clustering seen on the score plots, and were unique to the two plant cell lines.

From the results it is evident that PCA score plots suffice the understanding of apparent clustering/separation of samples due to their biochemical background. However, PCA is not capable of showing the underlying degree of similarities between the different clusters and hence the trend of responses within the data.

### Hierarchical cluster analysis

HCA, as a complimentary data reduction and pattern recognition method, was used for finding the underlying structure of objects through a repetitive process that associates (agglomerative methods) or dissociates (divisive methods) object by object until all are equally and completely processed (Downs and Barnard 2002;Steinbach et al. 2004). Automated HCA was performed on the data and the resulting dendrogram was calculated using the Ward linkage method (Ward 1963;Sato et al. 2008).

**Figure 2:**
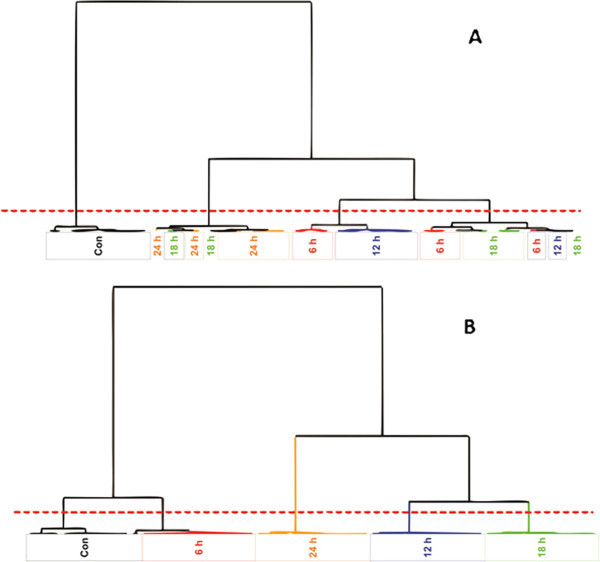
**HCA dendrograms showing the relationship between samples originating from INAP treated tobacco and sorghum cells at different time intervals**. The plot shows the relation between samples (**A**: tobacco and **B**: sorghum) as described by the length/distance of the node linking two clusters. The number of clusters can be deduced by counting the regions in which the dotted line crosses the node of each respective cluster.

The HCA dendrograms (Figure 2) show descriptively similar results to those of PCA: clustering of samples, with additional observation of the trend associated with the different time intervals, and the ordering of the samples’ grouping in relation to the time points. For instance, taking the tobacco HCA results into account (Figure 2A), a definitive clustering among the control samples can be seen. When the different treatment times are however considered, no definitive clustering exists and samples from the same treatment time groups are spread across four different clusters. The first cluster exclusively contains all the control samples. The second cluster is dominated by the 24 h samples and also contains some samples from the 18 h treatment time point. The third cluster is dominated by the 12 h treatment as well as some traces of samples from 6 h, and lastly, equal amounts of samples from 18 h and 6 h are seen in the fourth cluster which also contains a few samples from the 12 h time interval.

By comparison, the results obtained with sorghum samples show a very well structured response due to INAP treatment unlike tobacco, where maximum variation only exists between the control group and treatment samples as a whole. In sorghum, the five clusters representing extracts from different time points are well consolidated (Figure 2B). These depict the biological/treatment groups (control, 6 h, 12 h, 18 h, and 24 h). The first cluster exclusively contains samples from the control group, the second cluster contains samples from 6 h, the third contains samples from 24 h, the fourth cluster contains 12 h and the fifth group contains samples exclusively from the 18 h treatment time point. These results are indicative of more stringent metabolism of INAP by the sorghum cells in comparison to the tobacco cells, and suggests that the metabolic machinery of sorghum cells recognizes the oxime molecule more efficiently than that of tobacco cells, which shows variability across the different treatment time intervals. To get more insight into the statistical significance of differences (degrees of relatedness) in the clusters observed on PCA scores plots and HCA dendrograms, Metabolic Trees were computed.

### Metabolic trees and bootstrapping

**Figure 3:**
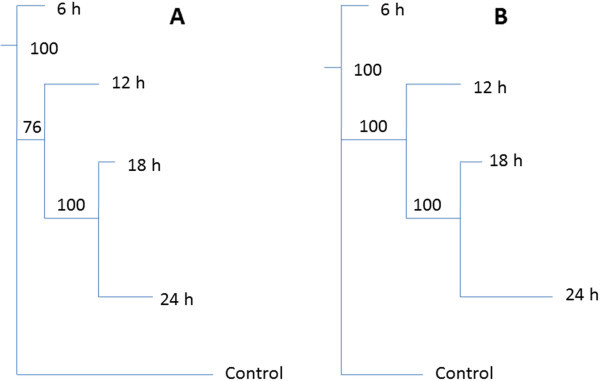
**Metabolomic Tree diagrams determined from the PCA scores plots of tobacco and sorghum samples**. The trees represent statistical evaluation of the degree of sample grouping and bootstrap numbers for each node are indicated on the tree diagram. **A**: tobacco and **B**: sorghum.

Metabolic Tree diagrams (PCA-to-Tree programme) allowed a statistical evaluation of the degree of sample grouping displayed by both PCA and HCA (Werth et al. 2010). During the generation of these trees, bootstrapping numbers, which determine the statistically significant differences between the different clusters, are also obtained (Halouska et al. 2012). Here, two independent tree diagrams corresponding to the tobacco and sorghum samples were generated using the data from the two dimensional PCA score plots. When comparing the trees (Figure 3), it can be seen that the distance between the clusters are different, due to different metabolic responses obtained from the two plant systems. Similarly to the HCA, the length of the lines (node) connecting the groups describes the distance between the clusters; for instance the horizontal line connecting the control and the rest of the groups is longer on the tobacco tree than it is on the sorghum tree. This is due to the fact that there is larger distance between the control group and the treated group on the tobacco data than for the sorghum system. Still, on both tree diagrams it is notable that the 18 h and 24 h extracts are closely related to each other, and this cluster appeared 100 times in both cases. Furthermore, the 18 h/24 h cluster is more closely related to the 12 h cluster than it is to the control and 6 h clusters. However, the cluster of 12 h/18 h/24 h appeared 100 times in sorghum and only 76 times on the tobacco system. This is consistent with the PCA results as it can be seen that the distance between the treatment times is smaller in tobacco than it is in the sorghum system. In deciphering the trends of these tree diagrams, it can be observed that the control is more closely related to the 6 h, followed by the 18/24 h and then lastly to the 12 h cluster. The fact that the bootstrapping values in the sorghum system were always 100 is evidence that there is a definite separation between the groups that is not as clear in the tobacco system. These observations are in line with those seen on the HCA.

### Shared and unique structures (SUS) plots

It is clear that PCA only evaluates global patterns (maximum variation) within the data and that better tools are required for understanding the differences between groups. For the same and other reasons stated by Van der Greef and Smilde (2005), alternative techniques have been proposed. Here, a supervised model, OPLS-DA (Trygg and Wold 2002), was used to reveal underlying responses which are associated with a time-trend (Shiryaeva et al. 2012) as shown by the HCA above. OPLS-DA can be considered as a modification of the traditional PLS-DA, with integral orthogonal signal correction filter (Bylesj et al. 2006;Wiklund et al. 2008;Smilde et al. 2010). The separation of ***Y*** -predictive (discriminating variation) and ***Y***-orthogonal variation (that which does not contribute to the class separation) facilitates the interpretability of the data, particularly in extracting information on changes in the molecular composition of samples. Thus, in this study, OPLS-DA was used to single out statistically and potentially significant biochemical variables (metabolites/biomarkers) responsible for differences among the various groups (classes represented by data from different time intervals). The OPLS-DA loadings plots, such as the S-plot and shared-and-unique-structures (SUS)-plot, enable the extraction of such statistically significant variables and identification of shared/unique structures in the samples (Wiklund et al. 2008). Although OPLS-DA is a very good statistical model, like other supervised models it also has some limitations, one being the possibility of over-fitting of the models. As such, supervised models need to be validated to ensure their significance. The results of such validation are presented as additional material (Additional file 1: Tables S1-S2).

**Figure 4:**
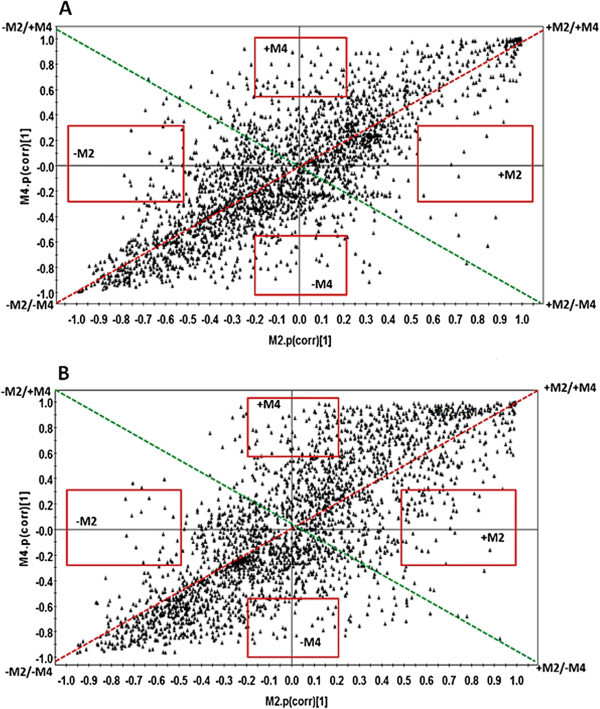
**Representative SUS-plots from independent OPLS-DA loadings S-plots**. This plot, constructed using a two correlation coefficient (p(corr)), shows how the metabolites from one independent model (Control vs. 6 h, M2) relates to those from the corresponding model (Control vs. 18 h, M4) for both tobacco **(A)** and sorghum **(B)**. The regions in which shared and unique metabolites reside are highlighted on the plot. The description of the different regions is given in the main text.

The use of SUS-plots was adapted in this study to decipher the differences in metabolic profiles obtained at different times following elicitation. OPLS-DA models were generated by comparing control and treated samples represented by each time interval. From each cell line, six different plots (6 h vs. 12 h, 6 h vs. 18 h, 6 h vs. 24 h, 12 h vs. 18 h, 12 h vs. 24 h, 18 h vs. 24 h) were generated and compared to each other. These combinations were derived from the respective loadings S-plots generated from the four different models, Control vs. 6 h (M2), Control vs. 12 h (M3), Control vs. 18 h (M4), Control vs. 24 h (M5) (data not shown). From the results, the SUS-plot was found to be more complementary to the HCA, since the same pattern can also be drawn from both. Figure 4 shows the SUS-plots generated by comparing M2 and M4 (6 h vs. 18 h), from both sorghum and tobacco. These two time points were chosen as they represent different stages of responses: the 6 h (M2) represent an early response and 18 h (M4) represent a mid to late response. Here, it can be seen that metabolites (*m/z* ions) in the tobacco model are more positively correlated and less scattered than in the sorghum model at the same time points. For instance, all the “shared structures”, i.e. the metabolites scattered across the red dotted line represent those which are positively correlated (++/--) and those scattered across the green dotted line represent those which are negatively correlated (+-/-+). Those which are found in the red boxes across the plot axes are either increasing (+M) or decreasing (-M) for that particular model and represent the “unique structures”. It is also important to note that metabolites which are on the extreme ends (outliers) of the axes contribute more significantly than those close to the center. Still, on the M2 vs. M4 tobacco SUS-plot, it can be seen that the distribution of the metabolites seems to create a latent line across the positively correlated diagonal line. The same is seen in the case of sorghum but is less pronounced as more metabolites are spread over the plot, especially on the positive side of both M2 and M4. It is such spreading which shows less “sharing” of metabolites in the sorghum vs. the tobacco models, hence a distinctive metabolic phenotype. When all the SUS-plots are considered (Additional file 1: Figures S1 and S2), it is evident that there is a tighter distribution of variables/metabolites across the different time points obtained from the tobacco cells compared to that of the sorghum cells. This observation strongly supports the hypothesis that oximes are more effectively/extensively metabolized in sorghum than in tobacco.

### Deriving biochemical insights from different models

By its own definition, metabolomics recognizes that the biological phenomena can only be characterized by the interrelationships of hundreds/thousands of variables simultaneously, and the choices for data analyses should be driven by the biological question, the data generating process, the experimental design and the assumptions of the data analysis methods (Kopka et al. 2004;Weckwerth and Morgenthal 2005;Smilde et al. 2010;Theodoridis et al. 2011). In general, MVDA methods focus on the associations between metabolites and their orchestrated or complementary behavior in relation to biological processes (Saccenti et al., 2014). The current study represents an adaptation of several MVDA approaches which highlights the use of traditional statistical visualization techniques to decipher the biological understanding of oxime metabolism in different plant systems and to display it for interpretation purposes.

We previously reported that INAP is recognized in tobacco cells by the enzymatic machinery of the phenylpropanoid pathway and bioconverted to a molecule, 4′-hexopyranosyloxy-3-methoxyisonitrosoacetophenone, with a substitution pattern similar to ferulic acid (Madala et al. 2012a). The same bio-conversion event was also detected in sorghum cells (data not shown). Furthermore, in both cell lines, INAP treatment was positively correlated with the increased synthesis of metabolites known in the context of plant stress - and defense responses. Results of tobacco extracts indicate that INAP affects the shikimate -, phenylpropanoid - and flavonoid pathways. Metabolites, tentatively annotated from the mass spectral data and online databases, included benzoic - or cinnamic acid derivatives that are either glycosylated or quinilated as well as flavonoid derivatives (Madala et al. 2013a). In addition to the biotransformation product, 4′-hexopyranosyloxy-3-methoxyisonitrosoacetophenone, preliminary annotation indicates that the sorghum metabolites accumulating in response to INAP treatment also include cinnamic - and benzoic acid derivatives and flavonoids (Table 1).

**Table 1:** **List of annotated bio-markers with tentative identification, representative of different metabolite classes, associated with response of (A) tobacco cells and (B) sorghum cells in response to treatment with 1 mM INAP**

A	Metabolite name	Core structure
**1**	4′-Hexopyranosyloxy-3-methoxyisonitrosoacetophenone,	INAP
**2**	1,2,4-Benzenetriol; 2-Me ether, 1-*O*-[3,4,5-trihydroxybenzoyl-(-> 6)-β-D-hexopyranoside]	Benzoic (gallic) acid
**3**	Quinic acid; (-)-form, 4-*O*-(4-hydroxy-3,5-dimethoxybenzoyl)	Benzoic (syringic) acid
**4**	3,4-Dihydroxybenzoic acid; 3-Me ether, 4-*O*-β-D-hexopyranoside	Benzoic (vanillic) acid
**5**	3-*O-*Caffeoylquinic acid	Cinnamic (caffeic) acid
**6**	4-*O*-beta-D-Hexopyranosyl-sinapate	Cinnamic (sinapic) acid
**7**	Kaempferol 3-rhamnosyl-(1- > 2)-hexopyranosyl-7-hexopyranose	Flavonoid
**B**	**Metabolite name**	**Core structure**
**1**	4*'*-Hexopyranosyloxy-3-methoxyisonitrosoacetophenone	INAP
**2**	Dhurrin	Cyanogenic glycoside
**3**	Trihydroxybenzophenone	Benzoic acid
**4**	4-*O*-Syringoylquinic acid	Benzoic (syringic) acid
**5**	Rhamnosyl, 3-*O*-(-methoxy-cinnamoyl)-3-acetyl	Cinnamic acid
**6**	Feruloyltyramine	Cinnamic (ferulic) acid
**7**	4*'*,5-Dihydroxy-7-prenylflavanone	Flavonoid

In addition to the global visual and qualitative representation of samples clustering shown by PCA, the computed HCA dendrograms highlighted visually the differential responses over time, suggesting thus time-dependent clustering/metabolic patterns with the data. The degree of relatedness of these sample groups could be assessed using the Metabolic Trees. The OPLS-DA SUS-plots indicated shared and unique variables from different clusters (time point samples), explaining further the different metabolite profile patterns observed.

Thus, the results from the complementarity of different computed models demonstrate that the two plant systems managed to recognize INAP, metabolize it, and that the biochemical profile is re-adjusted to internal equilibrium over time. The chemometric analyses of tobacco vs. sorghum results show the response in sorghum to be more uniform as compared to tobacco where a more variable response was obtained. It seems that INAP as a xenobiotic oxime, is more efficiently metabolized by cyanogenic as opposed to non-cyanogenic plants.

Biochemically, sorghum is a cyanogenic plant which is able to metabolize oxime containing precursors (Bak et al. 2000). INAP is an oxime molecule similar to intermediates/precursors during the biosynthesis of glucosinolates and cyanogenic glycosides, two classes of molecules that play vital roles during plant: pathogen/herbivore interactions (Neilson et al. 2013). Plants capable of metabolizing oxime precursors that are subsequently used for defense responses include sorghum and Arabidopsis (Bak et al. 2000), but not tobacco. The enzymes which code for the synthesis of cyanogenic glycosides and glucosinolates exist in tightly associated complexes or metabolons (Møller 2010). The finding of dhurrin as a signatory bio-marker in sorghum cells responding to INAP indicates that the metabolon for oxime metabolism is functional under these conditions. The coordinated response as seen in the MVDA of the sorghum cell extracts is thus a reflection of the system’s ability to recognize and metabolize oxime intermediates.

The existence of oximes in non-oxime metabolizing plants has been reported (Dubery et al. 1999) and suggests a possible role in plants other than defense (Madala et al. 2012a). In other plants the same set of enzymes might exist as well, but are found as a loosely associated metabolon and sometimes not all are present, as for tobacco. In the latter case, oxime precursors do not result in the accumulation of cyanogenic glycosides or related metabolites, but would rather be metabolized to amides and amines (Neilson et al. 2013).

In conclusion, the study extends our knowledge of the metabolism of oximes in plants, especially those that do not possess the biosynthetic ability generated by cyanogenic glucoside or glucosinolate metabolons. Furthermore, the use of PCA, HCA, Metabolic Trees and OPLS-DA-based SUS-plots in understanding the underlying pattern of biological responses at metabolic level is presented here. All these models clearly managed to show the superficial trend of INAP conversion over time and the associated metabolic changes which are intrinsic within the metabolomic data generated from the two compared plant systems. The use of these models as parallel approaches thus complements each other to uncover distinctive underlying trends that contribute additional insights into the biochemical events taking place.

## Material and methods

### Cell culture, treatment and metabolite extraction

*Nicotiana tabacum* cv ‘Samsun’ and *Sorghum bicolor* L. Moench cv ‘Sweet white’ cell suspensions were cultured as previously described (Gerber and Dubery 2003;Sanabria and Dubery 2006;Ngara et al. 2008). Three days after sub-culturing, aliquots (20 mL suspensions) were treated with 250 mM isonitrosoacetophenone (INAP), dissolved in acetone, to a final concentration of 1 mM with continuous rotation at 80 rpm and 25°C for 6, 12, 18, and 24 h time intervals. Control cells received no treatment. For the experimental design, a minimum of ten replicates for each biological group was utilized. After elicitation, cells were collected by means of vacuum filtration and metabolites extracted from the wet cells (2 g) by homogenization in 20 mL 100% methanol. To aid maximum extraction, the homogenates were allowed to agitate on a rotary shaker for at least 1 h. Cell debris was removed by means of centrifugation at 5000 × g for 10 min. The resulting supernatant was transferred to a new tube and the volume reduced to 1 mL with the aid of a Buchi rotary evaporator operating at 45°C, followed by drying to completeness in a microcentrifuge tube using a speed vacuum centrifuge operating at 45°C. The resulting pellet (from 2 g of cells) was dissolved in 400 μL 50% methanol and filtered through a 0.22 μm filter into a new UHPLC glass vial fitted with a 0.1 mL insert.

### Chromatographic- and mass spectrometric conditions

Chromatographic and mass spectrometric conditions were adapted and optimised from our previous work (Madala et al. [Bibr CR18][Bibr CR19][Bibr CR20][Bibr CR21]). Briefly, methanol extracts (5 μL) were analyzed on a Synapt UHPLC-high definition MS instrument (Waters, Corporation, USA) equipped with an Acquity BEH C18 column (100 mm × 2.1 mm with particle size of 1.7 μm) (Waters Corporation, USA). Two technical replicates for 5 independent samples were performed resulting in 10 injections for each biological group (control, 6, 12, 18, and 24 h). The composition of mobile phase A consisted of 0.1% formic acid in deionized water and mobile phase B consisted of 0.1% formic acid in methanol. The column was eluted with a linear gradient at a constant flow rate of 400 μL min^-1^ of 5% B over 0.0–2.0 min, 5–95% B over 2.0–22.0 min, held constant at 95% B over 22.0–25.0 min, 95–5% B over 25.0–27.0 min and a final wash at 5% B over 27–30 min. For MS acquisition, data was collected on a centroid mode and negative polarity electro-spray ionization (ESI) with a collision energy of 3 eV. Instrumental settings were as follows; capillary voltage: 2.5 kV, sample cone voltage: 17 V, extraction cone voltage: 5.0 V, MCP detector voltage: 1600 V, source temperature: 120°C, desolvation temperature: 350°C, cone gas flow: 50 (L h^-1^), desolvation gas flow: 450 L h^-1^), *m/z* range: 100–1000, scan time: 0.1 sec, interscan delay: 0.02 sec, lockmass: leucine enkephalin (556.3 μg mL^-1^), lockmass flow rate: 0.4 mL min^-1^, mass accuracy window: 0.5 Da.

### Data analyses

Primary data was further analyzed by MarkerlynxXS™ software (Waters Corporation, Milford, USA) for alignment, peak finding, peak integration and Rt correction with parameters as follows: retention time range (Rt) of 1–27 min, mass range of 100–1000 Da, mass tolerance of 0.02 D, Rt window of 0.2 min and, furthermore, isotopic peaks were excluded from the analysis. Peaks corresponding to INAP and its bio-conversion product were not included in the data analysis. Data was normalized to total intensity (area) using Markerlynx. The datasets thus obtained were exported to the SIMCA-P software version 12.0 (Umetrics, Umea, Sweden) in order to perform PCA and OPLS-DA. Before performing these multivariate data analyses, data was mean centered and Pareto-scaled for both models. For unsupervised models, the OPLS-DA based SUS-plots, cross-validated (CV)-Anova (SIMCA-P 12) was used to evaluate the over-fitting thereof (Additional file 1: Tables S1-S2).

In order to evaluate the effect of time on the response, HCA was automatically calculated and the resulting dendrogram evaluated with the aid of the SIMCA-P software. For HCA analysis, the Ward distance algorithm was used to calculate the distance between the different generated clusters. Using the PCA-to-Tree programme (Werth et al. 2010), the metabolomic tree diagrams were created and the corresponding bootstrap values calculated to interpret the PCA clustering pattern. Unlike in the case of HCA, where the Ward method was used, these tree diagrams were generated using the Euclidean distances method between the clusters from the PCA scores plots (Figure 1). Here, the standard bootstrapping techniques were used to generate a set of 100 distance matrices by randomly re-sampling the cluster centers and Euclidean distances. The matrices were then used in the PHYLIP phylogenetic software package (http://www.phylip.com) (Retief 2000) to generate 100 tree diagrams and a consensus tree diagram. The numbers on the trees indicates the bootstrap values which describes the number of times each node was present in the set of 100 tree diagrams. Bootstrap numbers below 50% indicates insignificant separation between the clusters.

## Electronic supplementary material

Additional file 1: Figure S1: A-E: OPLS-DA based SUS-plots showing metabolite distribution from different time intervals of INAP elicited tobacco cell suspensions. For the code description M2 encodes 6 h, M3 12 h, M4 18 h, and M5 24 h. **Figure S2**. A-E: OPLS-DA based SUS-plots showing metabolite distribution from different time intervals of INAP elicited sorghum cell suspensions. For the code description M2 encodes 6 h, M3 12 h, M4 18 h, and M5 24 h. **Tables S1–S2**. Cross-validation (CV)-Anova of OPLS-DA-derived SUS plots for tobacco and sorghum. M2, M3, M4 and M5 refers to Control vs. 6 h, Control vs. 12 h, Control vs. 18 h and Control vs. 24 h respectively. (PDF 718 KB)
