# Supplementary material for: Multivariate statistical models of metabolomic data reveals different metabolite distribution patterns in isonitrosoacetophenone-elicited Nicotiana tabacum and Sorghum bicolor cells
Source: Springerplus. 2014 May 20;3:254. doi: 10.1186/2193-1801-3-254 (PMC4044000; doi:10.1186/2193-1801-3-254)
Supplement: Supplementary file 1 — Additional file 1: Figure S1: A-E: OPLS-DA based SUS-plots showing metabolite distribution from different time intervals of INAP elicited tobacco cell suspensions. For the code description M2 encodes 6 h, M3 12 h, M4 18 h, and M5 24 h. Figure S2. A-E: OPLS-DA based SUS-plots showing metabolite distribution from different time intervals of INAP elicited sorghum cell suspensions. For the code description M2 encodes 6 h, M3 12 h, M4 18 h, and M5 24 h. Tables S1–S2. Cross-validation (CV)-Anova of OPLS-DA-derived SUS plots for tobacco and sorghum. M2, M3, M4 and M5 refers to Control vs. 6 h, Control vs. 12 h, Control vs. 18 h and Control vs. 24 h respectively. (PDF 718 KB) [file 40064_2014_978_MOESM1_ESM.pdf]

Multivariate statistical models of metabolomic data reveals different metabolite distribution patterns in isonitrosoacetophenone-elicited *Nicotiana tabacum* and *Sorghum bicolor* cells.

Ntakadzeni E Madala[1], Lizelle A Piater[1], Paul A Steenkamp[1,2] and Ian A Dubery[1*].

[1]Department of Biochemistry, University of Johannesburg, Auckland Park, 2006, South Africa

[2]BioSciences, CSIR, Pretoria, 0001, South Africa.

<u>Additional file 1</u>:

Figure S1 A-E: OPLS-DA based SUS-plots showing metabolite distribution from different time intervals of INAP elicited tobacco cell suspensions. For the code description M2 encodes 6 h, M3 12 h, M4 18 h, and M5 24 h.

Figure S2 A-E: OPLS-DA based SUS-plots showing metabolite distribution from different time intervals of INAP elicited sorghum cell suspensions. For the code description M2 encodes 6 h, M3 12 h, M4 18 h, and M5 24 h.

Tables S1–S2. Cross-validation (CV)-Anova of OPLS-DA-derived SUS plots for tobacco and sorghum. M2, M3, M4 and M5 refers to Control vs. 6 h, Control vs. 12 h, Control vs. 18 h and Control vs. 24 h respectively.

Figure S1: OPLS-DA based SUS-plots showing metabolite distribution from different treatment time intervals, **A** (6 h vs12 h), **B** (6 h vs 24 h), **C** (12 h vs 18 h), **D** (12 h vs 24 h) and **E** (18 h vs 24) of **tobacco cell suspensions** treated with 1 mM isonitrosacetophenone. For keys features, refer to Fig. 4.

S1-A.

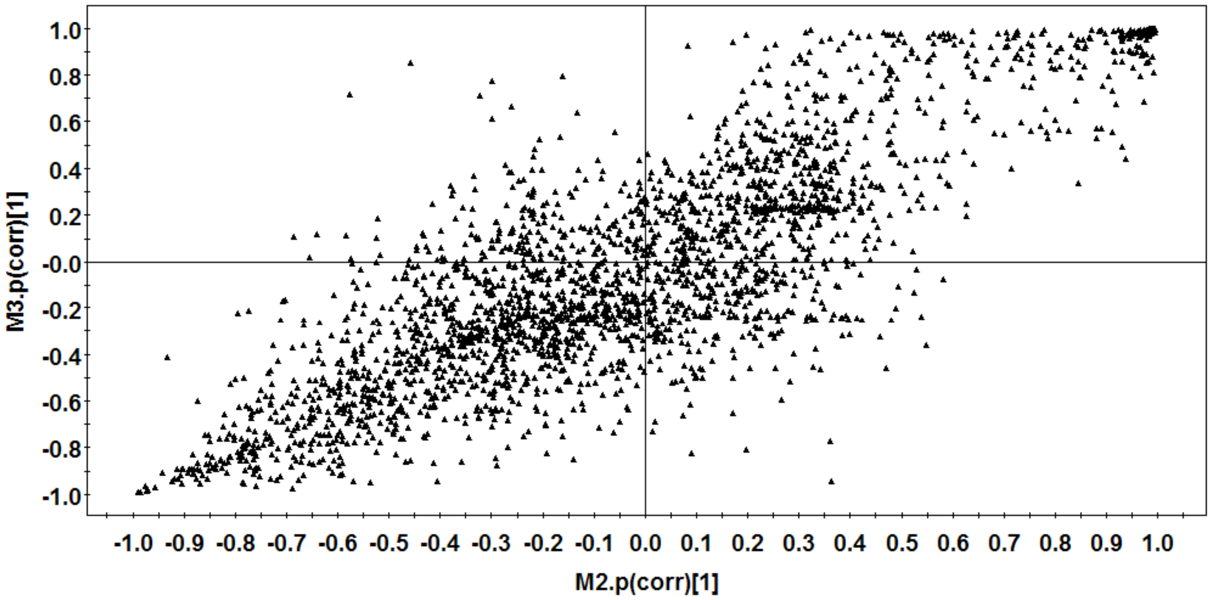

S1-B.

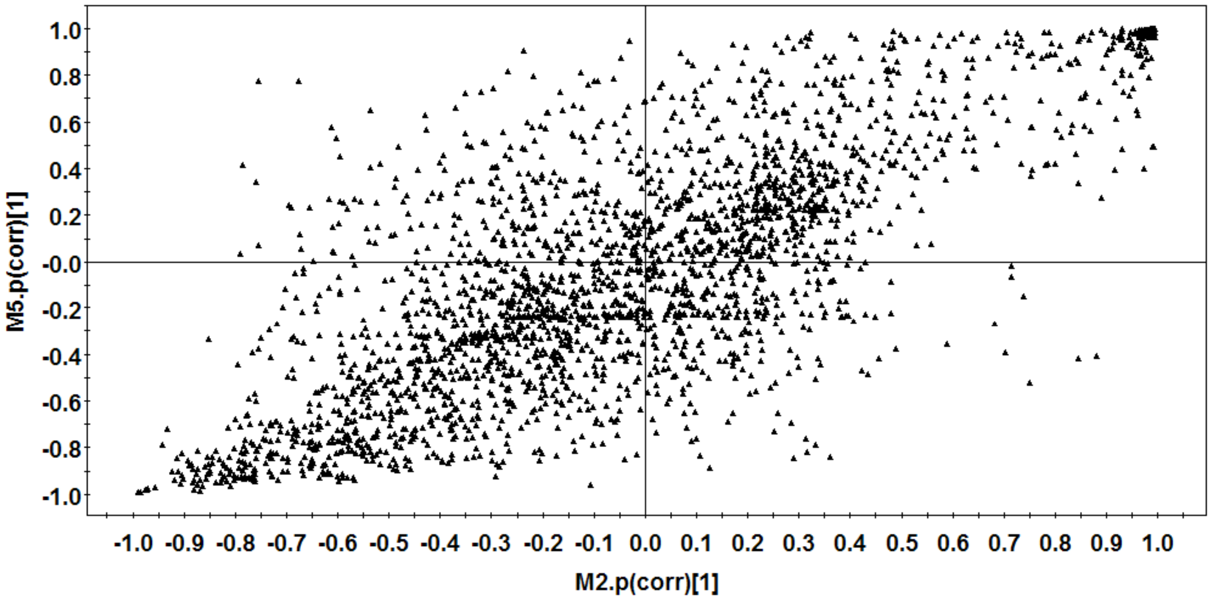

S1-C.

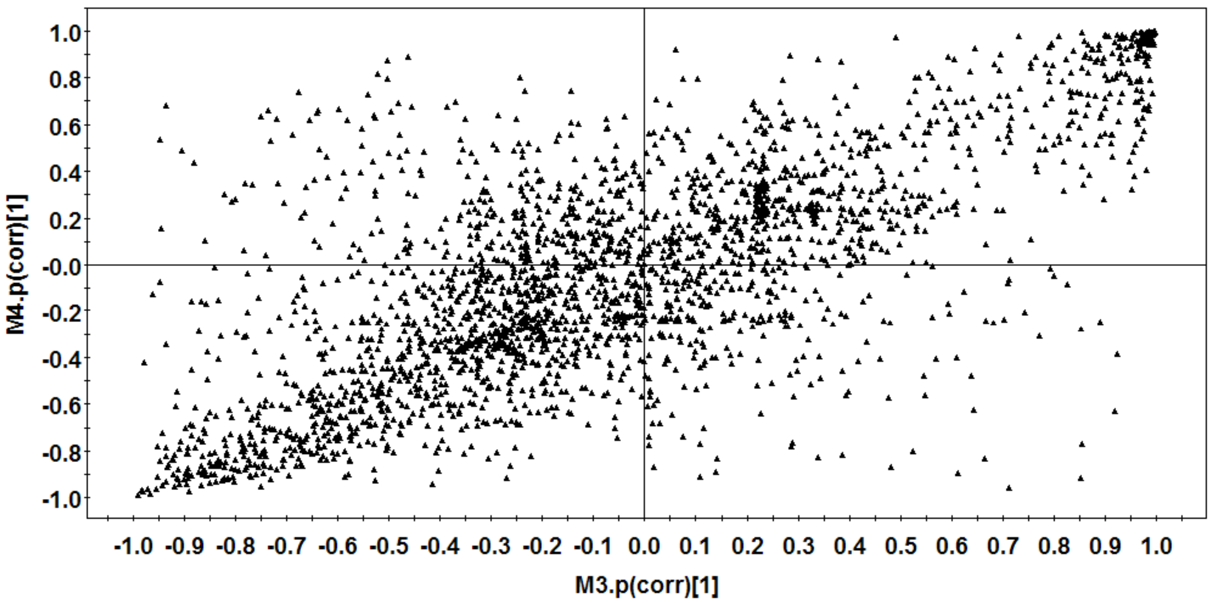
1.0
0.8
0.6
0.4
0.2
-0.0
-0.2
-0.4
-0.6
-0.8
-1.0
M4.p(corr)[1]
-1.0 -0.9 -0.8 -0.7 -0.6 -0.5 -0.4 -0.3 -0.2 -0.1 0.0 0.1 0.2 0.3 0.4 0.5 0.6 0.7 0.8 0.9 1.0
M3.p(corr)[1]

S1-D.

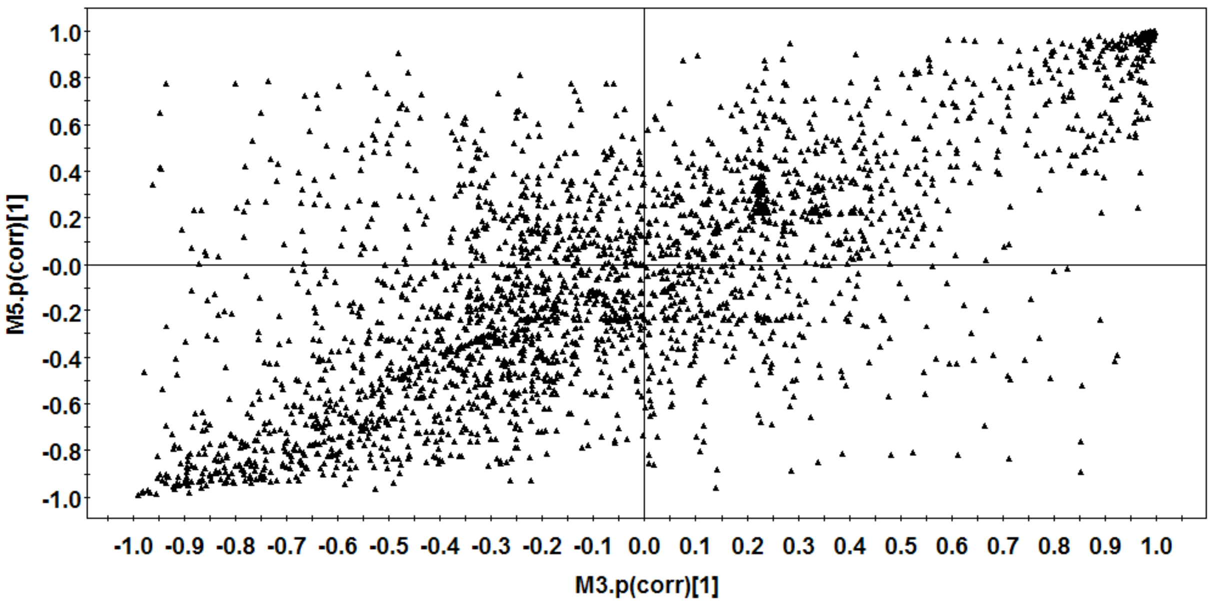
1.0
0.8
0.6
0.4
0.2
-0.0
-0.2
-0.4
-0.6
-0.8
-1.0
M5.p(corr)[1]
-1.0 -0.9 -0.8 -0.7 -0.6 -0.5 -0.4 -0.3 -0.2 -0.1 0.0 0.1 0.2 0.3 0.4 0.5 0.6 0.7 0.8 0.9 1.0
M3.p(corr)[1]

S1-E.

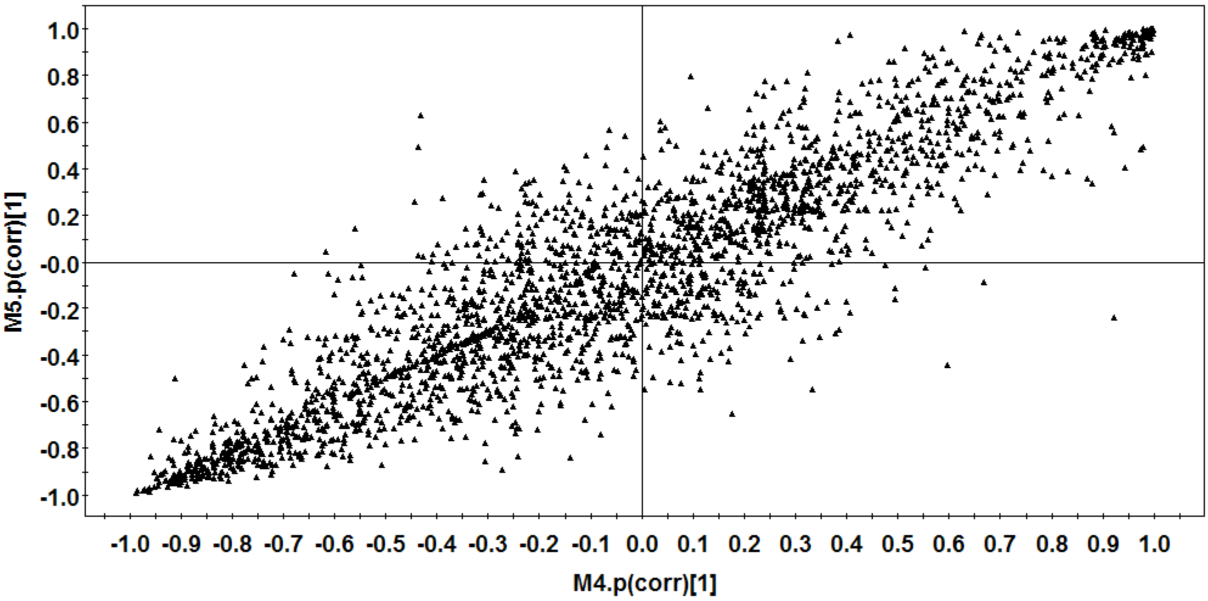
1.0
0.8
0.6
0.4
0.2
-0.0
-0.2
-0.4
-0.6
-0.8
-1.0
M5.p(corr)[1]
-1.0 -0.9 -0.8 -0.7 -0.6 -0.5 -0.4 -0.3 -0.2 -0.1 0.0 0.1 0.2 0.3 0.4 0.5 0.6 0.7 0.8 0.9 1.0
M4.p(corr)[1]

Figure S2: OPLS-DA based SUS-plots showing metabolite distribution from different treatment time intervals, **A** (6 h vs.12 h), **B** (6 h vs. 24 h), **C** (12 h vs. 18 h), **D** (12 h vs. 24 h) and **E** (18 h vs. 24) of **sorghum cell suspensions** treated with 1 mM isonitrosacetophenone. For keys features, refer to Fig. 4.

S2-A.

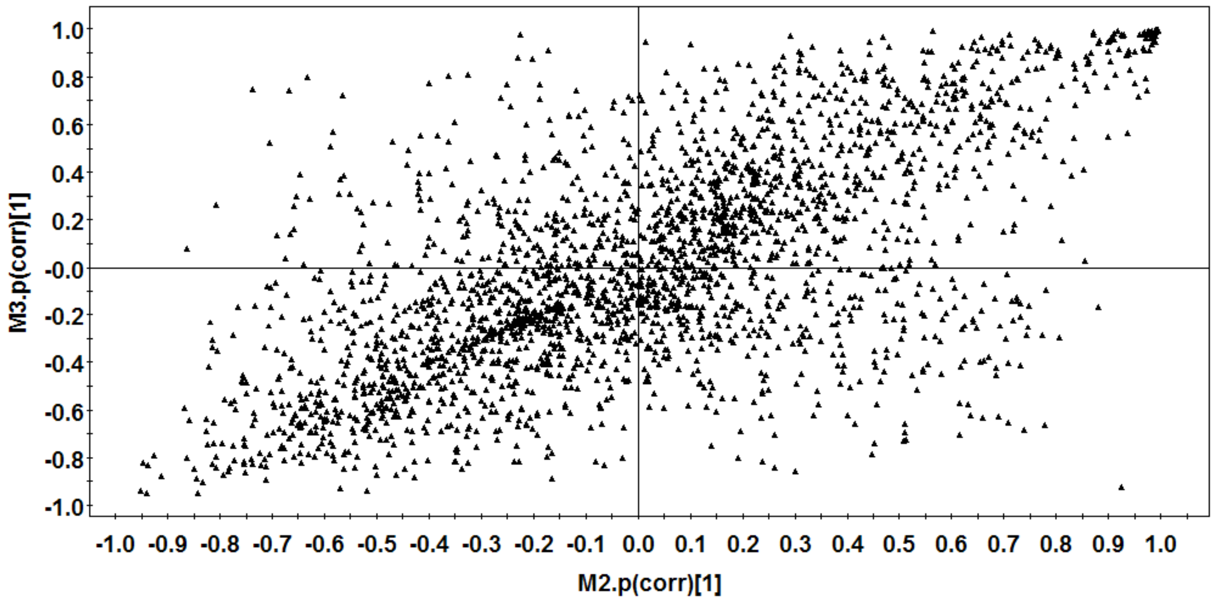

S2-B.

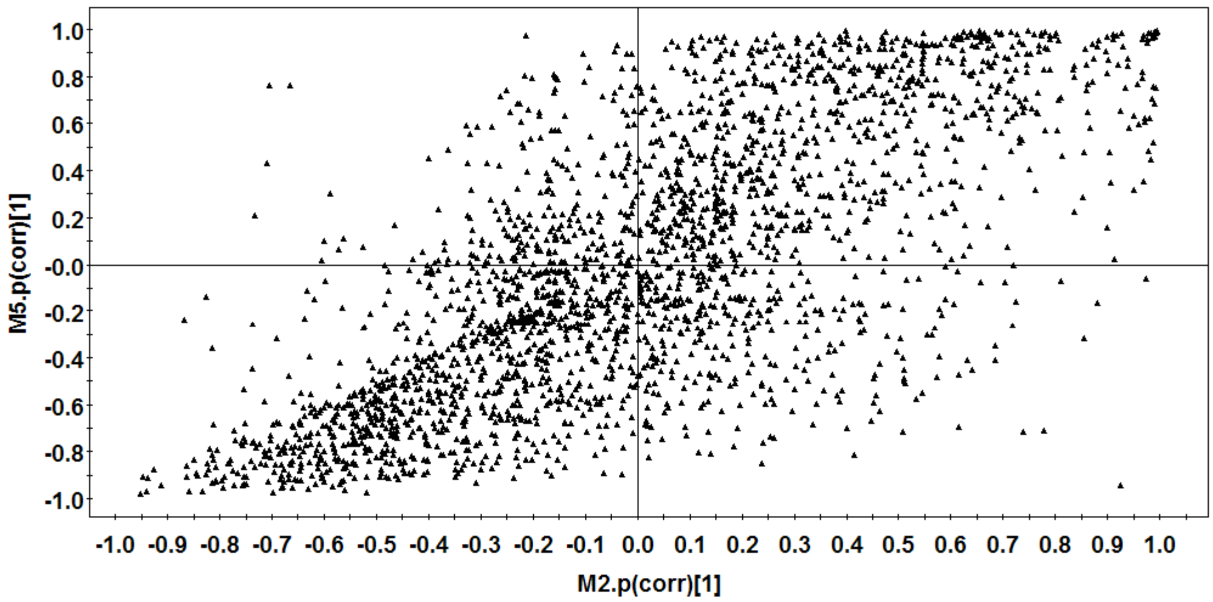

S2-C.

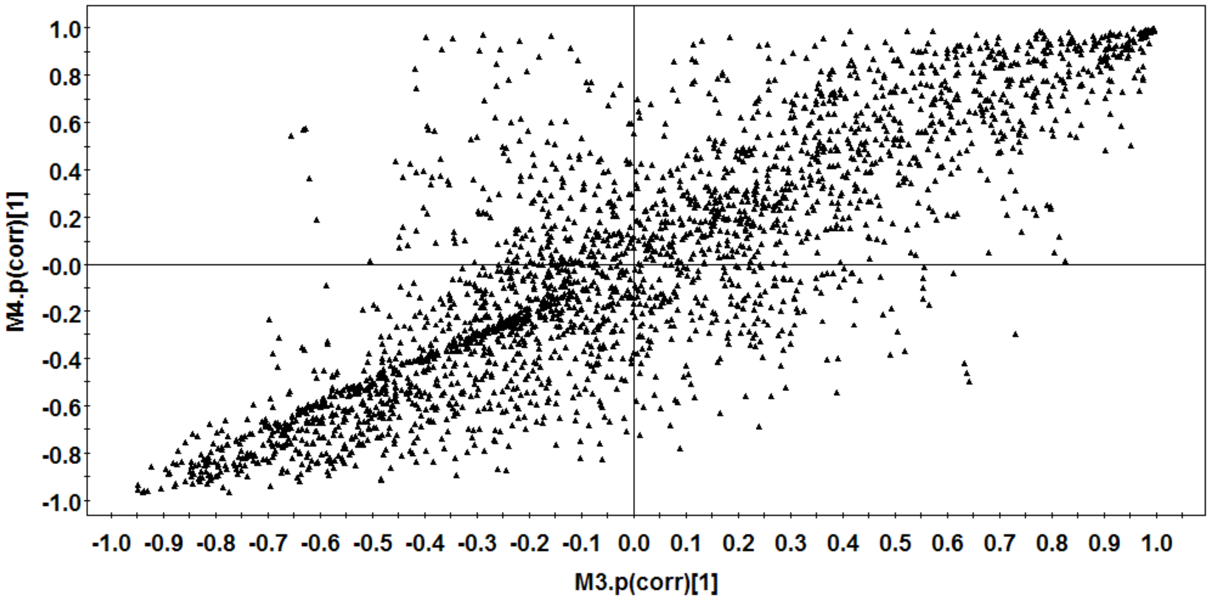
1.0
0.8
0.6
0.4
0.2
-0.0
-0.2
-0.4
-0.6
-0.8
-1.0
M4.p(corr)[1]
-1.0 -0.9 -0.8 -0.7 -0.6 -0.5 -0.4 -0.3 -0.2 -0.1 0.0 0.1 0.2 0.3 0.4 0.5 0.6 0.7 0.8 0.9 1.0
M3.p(corr)[1]

S2-D.

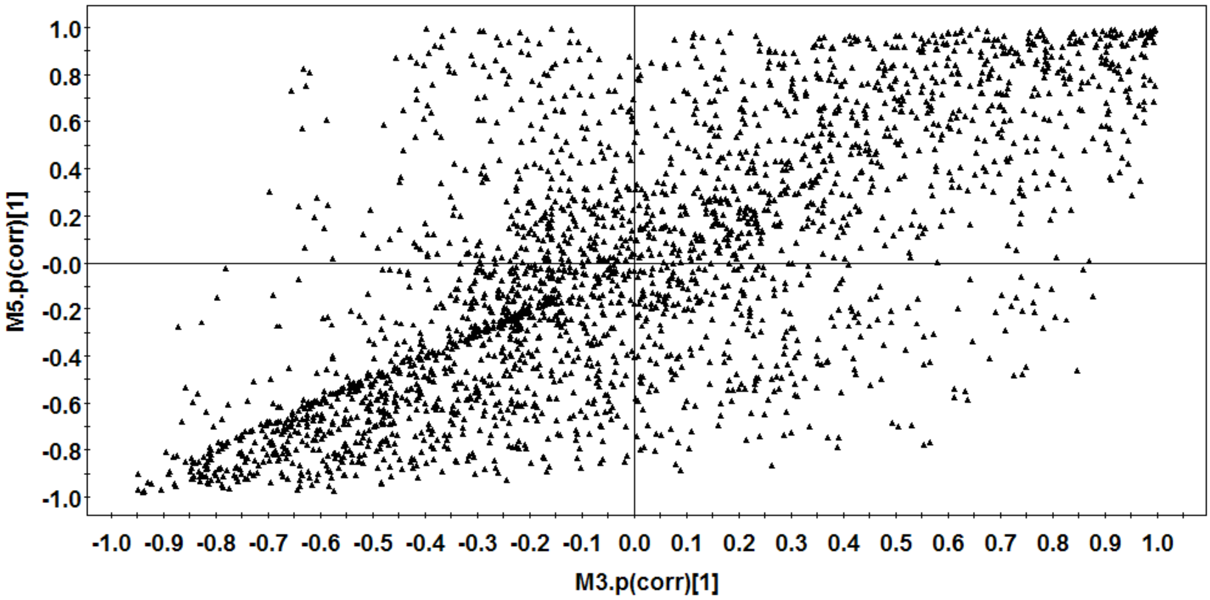
1.0
0.8
0.6
0.4
0.2
-0.0
-0.2
-0.4
-0.6
-0.8
-1.0
M5.p(corr)[1]
-1.0 -0.9 -0.8 -0.7 -0.6 -0.5 -0.4 -0.3 -0.2 -0.1 0.0 0.1 0.2 0.3 0.4 0.5 0.6 0.7 0.8 0.9 1.0
M3.p(corr)[1]

S2-E.

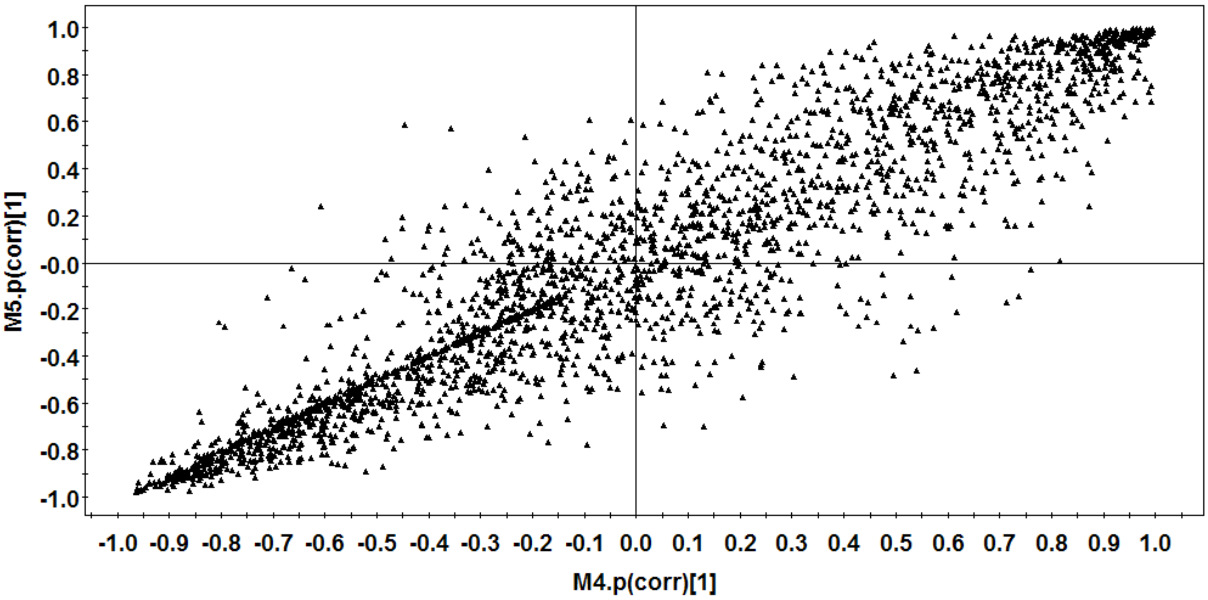

M5.p(corr)[1]
M4.p(corr)[1]
1.0
0.8
0.6
0.4
0.2
-0.0
-0.2
-0.4
-0.6
-0.8
-1.0
-1.0 -0.9 -0.8 -0.7 -0.6 -0.5 -0.4 -0.3 -0.2 -0.1 0.0 0.1 0.2 0.3 0.4 0.5 0.6 0.7 0.8 0.9 1.0

Tables S1 –S2. CV-Anova validation of OPLS-DA derived SUS plots. M2, M3, M4 and M5 refers to Control vs. 6 h, Control vs. 12 h, Control vs. 18 h and Control vs. 24 h respectively.

1. INAP treated tobacco cells

a. CV-anova results for the OPLS model for M2

[R^2X (cum) = 0.390; R^2Y (cum) = 0.995; Q^2 (cum) = 0.966].

**M2(Untitled)**	**SS**	**DF**	**MS**	**F**	**P**	**SD**
**Total corr.**	19	19	1			1
**Regression**	18.3612	4	4.59031	107.795	7.34028e-011	2.1425
**Residual**	0.638758	15	0.0425839			0.206359

b. CV-anova results for the OPLS model for M3

[R^2X (cum) = 0.493; R^2Y (cum) = 0.998; Q^2 (cum) = 0.985].

**M3(Untitled)**	**SS**	**DF**	**MS**	**F**	**P**	**SD**
**Total corr.**	18	18	1			1
**Regression**	17.7266	4	4.43165	226.929	1.4723e-012	2.10515
**Residual**	0.273403	14	0.0195288			0.139745

c. CV-anova results for the OPLS model for M4

[R^2X (cum) = 0.444; R^2Y (cum) = 0.998; Q^2 (cum) = 0.986].

**M4(Untitled)**	**SS**	**DF**	**MS**	**F**	**P**	**SD**
**Total corr.**	19	19	1			1
**Regression**	18.7399	4	4.68496	270.135	8.8637e-014	2.16448
**Residual**	0.260146	15	0.0173431			0.131693

d. CV-anova results for the OPLS model for M5

[R^2X (cum) = 0.486; R^2Y (cum) = 0.999; Q^2 (cum) = 0.985].

**M5(Untitled)**	**SS**	**DF**	**MS**	**F**	**P**	**SD**
**Total corr.**	19	19	1			1
**Regression**	18.7138	4	4.67846	245.239	1.80922e-013	2.16297
**Residual**	0.286157	15	0.0190772			0.13812

2. INAP treated sorghum cells

a. CV-anova results for the OPLS model for M2

[R^2X (cum) = 0.514; R^2Y (cum) = 0.998; Q^2 (cum) = 0.985].

M2(Con vs 6 h)	SS	DF	MS	F	p	SD
Total corr.	39	39	1			1
Regression	38.4257	6	6.40428	367.982	9.34485e-029	2.53067
Residual	0.574325	33	0.0174038			0.131923

b. CV-anova results for the OPLS model for M3

[R^2X (cum) = 0.498; R^2Y (cum) = 0.996; Q^2 (cum) = 0.992].

M3(Con vs 12 h)	SS	DF	MS	F	p	SD
Total corr.	39	39	1			1
Regression	38.6956	4	9.67389	1112.15	2.40803e-036	3.11029
Residual	0.304442	35	0.00869835			0.093265

c. CV-anova results for the OPLS model for M4

[R^2X (cum) = 0.550; R^2Y (cum) = 0.996; Q^2 (cum) = 0.993].

M4(Con vs 18 h)	SS	DF	MS	F	p	SD
Total corr.	39	39	1			1
Regression	38.7321	4	9.68303	1265.14	2.56831e-037	3.11176
Residual	0.267879	35	0.0076537			0.0874854

d. CV-anova results for the OPLS model for M5

[R^2X (cum) = 0.584; R^2Y (cum) = 0.997; Q^2 (cum) = 0.994].

M5(Con vs 24 h)	SS	DF	MS	F	p	SD
Total corr.	39	39	1			1
Regression	38.7779	4	9.69448	1527.85	0	3.1136
Residual	0.222081	35	0.00634516			0.0796565

Reference

L. Eriksson, J. Trygg, S. Wold, CV-ANOVA for significance testing of PLS and OPLS® models, J. Chemometr 22 (2008) 594-600.
